# The Role of High-Sensitivity Troponin T Regarding Prognosis and Cardiovascular Outcome across Heart Failure Spectrum

**DOI:** 10.3390/jcm13123533

**Published:** 2024-06-17

**Authors:** Andrea D’Amato, Paolo Severino, Silvia Prosperi, Marco Valerio Mariani, Rosanna Germanò, Andrea De Prisco, Vincenzo Myftari, Claudia Cestiè, Aurora Labbro Francia, Stefanie Marek-Iannucci, Leonardo Tabacco, Leonardo Vari, Silvia Luisa Marano, Gianluca Di Pietro, Carlo Lavalle, Gennaro Sardella, Massimo Mancone, Roberto Badagliacca, Francesco Fedele, Carmine Dario Vizza

**Affiliations:** 1 Department of Clinical, Internal, Anaesthesiology and Cardiovascular Sciences, Sapienza University of Rome, Viale del Policlinico 155, 00161 Rome, Italy; paolo.severino@uniroma1.it (P.S.); silviapro@outlook.it (S.P.); rosanna.germano@gmail.com (R.G.); deprisco.1843735@studenti.uniroma1.it (A.D.P.); vincenzo.myftari@gmail.com (V.M.); claudia.cestie@gmail.com (C.C.); auro1298@gmail.com (A.L.F.); stefanie.marekiannucci@gmail.com (S.M.-I.); tabacco.1852080@studenti.uniroma1.it (L.T.); leonardovari98@gmail.com (L.V.); silviamarano35@gmail.com (S.L.M.); gianluca.dipietro@uniroma1.it (G.D.P.); carlo.lavalle@uniroma1.it (C.L.); gennaro.sardella@uniroma1.it (G.S.); massimo.mancone@uniroma1.it (M.M.); roberto.badagliacca@uniroma1.it (R.B.); dario.vizza@uniroma1.it (C.D.V.); 2 Department of Cardiology, Ospedale Fabrizio Spaziani, 03100 Frosinone, Italy; 3 San Raffaele Cassino, 03043 Cassino, Italy; francesco.fedele@uniroma1.it

**Keywords:** heart failure, biomarkers, high-sensitivity cardiac troponin T, hospitalization, cardiovascular mortality, worsening heart failure

## Abstract

**Background:** Cardiac troponin release is related to the cardiomyocyte loss occurring in heart failure (HF). The prognostic role of high-sensitivity cardiac troponin T (hs-cTnT) in several settings of HF is under investigation. The aim of the study is to assess the prognostic role of intrahospital hs-cTnT in patients admitted due to HF. **Methods:** In this observational, single center, prospective study, patients hospitalized due to HF have been enrolled. Admission, in-hospital peak, and discharge hs-cTnT have been assessed. Patients were followed up for 6 months. Cardiovascular (CV) death, HF hospitalization (HFH), and worsening HF (WHF) (i.e., urgent ambulatory visit/loop diuretics escalation) events have been assessed at 6-month follow up. **Results:** 253 consecutive patients have been enrolled in the study. The hs-cTnT median values at admission and discharge were 0.031 ng/mL (IQR 0.02–0.078) and 0.031 ng/mL (IQR 0.02–0.077), respectively. The risk of CV death/HFH was higher in patients with admission hs-cTnT values above the median (*p* = 0.02) and in patients who had an increase in hs-cTnT during hospitalization (*p* = 0.03). Multivariate Cox regression analysis confirmed that hs-cTnT above the median (OR: 2.06; 95% CI: 1.02–4.1; *p* = 0.04) and increase in hs-cTnT during hospitalization (OR:1.95; 95%CI: 1.006–3.769; *p* = 0.04) were predictors of CV death/HFH. In a subgroup analysis of patients with chronic HF, hs-cTnT above the median was associated with increased risk of CV death/HFH (*p* = 0.03), while in the subgroup of patients with HFmrEF/HFpEF, hs-cTnT above the median was associated with outpatient WHF events (*p* = 0.03). **Conclusions:** Inpatient hs-cTnT levels predict CV death/HFH in patients with HF. In particular, in the subgroup of chronic HF patients, hs-cTnT is predictive of CV death/HFH; while in patients with HFmrEF/HFpEF, hs-cTnT predicts WHF events.

## 1. Introduction

Heart failure (HF) is one of the main causes of morbidity and mortality worldwide [1]. It is a clinical syndrome caused by the incapacity of the heart to maintain normal systemic perfusion at normal intraventricular filling pressures. Once diagnosed, patients with HF have an average rate of one hospital readmission per year [1,2] and an estimated mortality rate of 67% within five years [3].

HF is characterized by variable periods of symptomatic stability, often interrupted by episodes of decompensated HF despite optimized therapy. The phases of clinical deterioration are increasingly recognized as a distinct phase in the history of HF, termed worsening HF (WHF) [4]. WHF is a condition of deterioration of clinical signs of HF, despite optimized medical management, requiring escalation of diuretic therapy, hospitalization or urgent ambulatorial visits [5]. The interesting and challenging aspect of this condition is that the culminating event of WHF is hospitalization, but the progressive worsening develops outside of the hospital, and it is often subclinical, manifesting itself with myocardial biomarkers increase, need for diuretic escalation, as well as symptoms and signs requiring urgent observation by a cardiologist in the outpatient setting. The early identification of patients in need of diuretic dose adjustments and ambulatory urgent visits may be crucial in the management of these patients in order to avoid hospitalization and related adverse events.

Besides echocardiographic parameters, natriuretic peptides (NPs) are fundamental to rule out the clinical condition of HF and to predict short-term mortality in patients hospitalized due to the latter [1,2]. The association between NPs and poor prognosis has been demonstrated [6]. High pre-discharge levels of brain natriuretic peptide (BNP) and N-terminal pro B-type natriuretic peptide (NT-proBNP) are associated with a high risk of cardiovascular (CV) death and hospital readmission [7]. Similar findings have been reported in the OPTIMIZE-HF registry [8]. In acute HF, congestion is the main factor influencing NP elevation. However, in chronic stable conditions, transmural wall stress is usually the main determinant of NP concentrations. On the other hand, the mechanism behind NP augmentation in HF with preserved ejection fraction (HFpEF) is less clear, since there is a reduction in wall stress due to the generally smaller size of the left ventricular chamber. Comorbidities, such as kidney disease or obesity, may also affect the concentration of NPs and thus the prognostic significance of these biomarkers [9].

NPs are sensitive prognostic markers in HF, but it may be important to identify alternative biomarkers for more accurate management and prognostic stratification of HF patients. Recently, the importance of the high-sensitivity cardiac troponin T (hs-cTnT) assay in the diagnosis and prognosis of HF has been demonstrated [10]. Troponins are part of the skeletal and cardiac myocyte contraction system. Different troponin isoforms are represented in the different muscle types. While troponin C is synthesized in equal manner in skeletal and cardiac myocytes, the troponin T and I isoforms are highly specific [11]. The latter are expressed especially in cardiac myocytes and are by far the most specific and sensitive indicators for the diagnosis of acute myocardial infarction (AMI) [12]. Myocyte damage induces troponin release into the circulation. The increase in hs-cTnT levels is directly related to the severity of myocyte damage, making troponins quantitative markers of heart tissue damage [13]. Molecular events such as cardiomyocyte death and apoptosis also take place during chronic disease, and high hs-cTnT levels are representative of the long-standing cardiac damage occurring in HF. In fact, in patients with dilatative cardiomyopathy, higher hs-cTnT levels were found to be predictive of a deterioration in clinical conditions [14]. Setsuka et al. [15] have shown that higher troponin levels are found in severe HF, with advanced New York Heart Association (NYHA) class, and in patients who developed complications and HF exacerbation. Various studies have investigated the predictive power of troponin levels in HF patients, showing a higher incidence of major CV events in patients with higher troponin levels [16]. These studies mainly included patients with HF with reduced ejection fraction (HFrEF) and had major CV events as their main endpoints. Evidence regarding the role of cardiac troponins in HF subpopulations is lacking. Furthermore, the prognostic role of cardiac troponins in terms of WHF events (i.e., the need for diuretic escalation or urgent ambulatory visits due to HF) has not been investigated yet.

The aim of the current study was to assess the role of inpatient cardiac hs-cTnT regarding the identification of HF patients at higher risk of adverse events, including CV mortality, HFH and WHF events, with special focus on the different subgroups of HF.

## 2. Methods

This was an observational, prospective, single center study, enrolling patients with a diagnosis of HF who have been consecutively admitted to the Department of Clinical, Internal, Anesthesiology and Cardiovascular Sciences at Policlinico Umberto I, Sapienza University of Rome. Inclusion criteria were the following: (I) written, signed and dated informed consent; (II) age above 18 years; (III) diagnosis of HF according to the Guidelines [1]. Exclusion criteria were the following: (I) presence of any condition representing the main cause of hs-cTnT increase beyond HF; (II) planned or history of heart transplantation or ventricular assist device (VAD); (III) end-stage kidney failure and/or dialysis; (IV) any condition limiting life expectancy less than one year; (V) pregnancy or nursing; (VI) non-compliance with the study protocol.

Patients enrolled constituted one study group.

The following parameters were collected: (i) clinical parameters (past medical history, physical examination, electrocardiogram, arterial blood pressure, NYHA class, and pharmacological therapy); (ii) echocardiographic parameters (ventricular chambers size, systolic and diastolic function, and valve disease and severity); (iii) laboratory parameters (hs-cTnT, blood cell count, creatinine, electrolytes, alanine aminotransferase, and aspartate aminotransferase). Specifically, the admission, peak, and discharge values of hs-cTnT were recorded. Moreover, the delta between admission and peak values of hs-cTnT was calculated. An increase in hs-cTnT during an in-hospital stay was defined as a delta of at least 0.014 ng/mL, between the admission and peak hs-cTnT values (representing the upper reference limit of hs-cTnT). The assay Elecsys^®^ (Roche Diagnostics International Ltd., Rotkreuz, Switzerland) for hs-cTnT has been used.

Over a follow-up period of 6 months after the index hospitalization, CV death, HFH, and urgent ambulatory visits/need of loop diuretic escalation were investigated in the outpatient HF clinic.

Specific subgroup analyses according to LVEF values and clinical presentation of HF were performed in order to define the prognostic role of hs-cTnT in terms of CV death, HFH, and urgent ambulatory visits/need of loop diuretic escalation.

Data were collected in a dedicated Excel Database (Version 2405 Build 16.0.17628.20006; 64 bit). The study was conducted according to the Helsinki Declaration. The study protocol was approved by the Ethical Committee of Policlinico Umberto I in Rome (rif.7068, approved on 8 May 2023).

### Statistical Analysis

The normal distribution of continuous variables was assessed with the Kolmogorov–Smirnov test. Continuous variables were expressed as mean and standard deviation, whereas median and first and third quartiles were used for non-normally distributed data. Categorical data were described as numbers and percentages. Student’s *t*-test, the Mann–Whitney test, the χ^2^ test, and the Fisher exact test were used for comparisons, as needed. The Kaplan–Meier method was used to estimate the cumulative event rates of study outcomes in the overall population, categorized based on admission troponin (above or below the median value of the studied population) and on the basis of the trend in troponin values during the hospitalization (patients with an increase or decrease in troponin values). Kaplan–Meier analysis was used to analyze the differences in clinical outcome rates in subgroups of patients with HFpEF and HF with mildly reduced ejection fraction (HFmrEF), and in patients with chronic HF presentation. The differences in each group were compared using log-rank tests. Univariate and multivariate Cox regression analyses were performed to obtain the odds ratios (ORs) of the associations among hs-cTnT with the endpoints. All the associations among variables and the composite endpoints with a *p*-value < 0.1 at univariate analysis were included at multivariate analysis. At multivariate analysis, variables potentially associated with the composite outcomes of CV death and HFH have been considered. For all tests, a *p*-value < 0.05 was considered statistically significant.

The statistical analysis was performed using SPSS version 27.0 for Mac (IBM Software, Inc., Armonk, NY, USA).

## 3. Results

A total of 253 consecutive patients were enrolled from October 2022 to April 2023 and they were followed-up for a period of 6 months.

**Figure 1:**
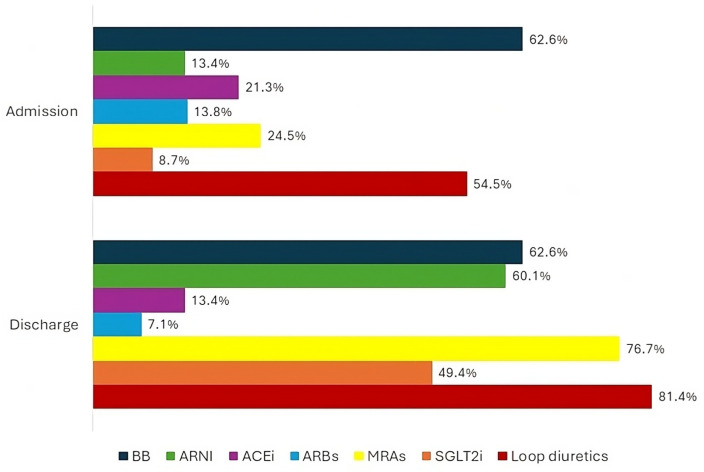
Percentage of total population on treatment with heart failure disease modifying drugs and loop diuretics at hospital admission and discharge. HF—heart failure; BB—beta blocker; ARNI—angiotensin receptor/neprilysin inhibitor; ACEi—angiotensin-converting enzyme inhibitor; ARBs—angiotensin receptor blockers; MRAs—mineralocorticoid receptor antagonists; SGLT2i—sodium glucose cotransporter 2 inhibitor.

**Figure 2:**
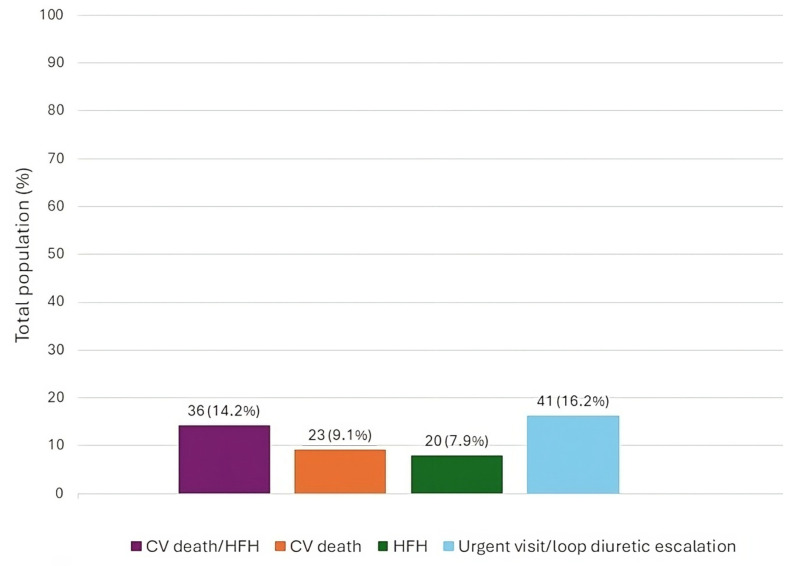
Total number and percentage rate of adverse events in the total population at 6-month follow-up. CV—cardiovascular; HFH—heart failure hospitalization.

The baseline features of the patient population are listed in Table 1. The types of admission and discharge therapies for the total population have been represented in Figure 1. The occurrence of each outcome in the total population has been represented in Figure 2.

Considering the total population, the composite of CV death and HFH was significantly higher in patients with hs-cTnT levels at admission above the median (23 vs. 13; 19.8% vs. 9.5%; *p* = 0.02) and in patients with a significant increase in hs-cTnT during hospitalization (20 vs. 16; 20% vs. 10.5%; *p* = 0.03) (Table 2 and Table 3).

**Figure 3:**
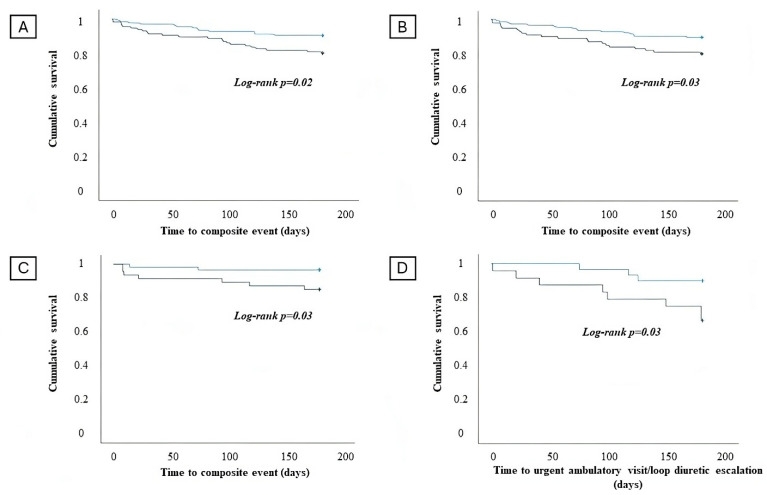
Survival analysis regarding the occurrence of the composite of cardiovascular (CV) death and heart failure hospitalization (HFH) in patients with an admission high-sensitivity T troponin (hs-cTnT) value below the median (blue line) and admission high-sensitivity T troponin value above the median (green line) in the overall population (**A**). Survival analysis regarding the occurrence of the composite of CV death and HFH in patients without significant in-hospital hs-cTnT increase (blue line) and with significant hs-cTnT increase (green line) in the overall population (**B**). Survival analysis regarding the occurrence of the composite of CV death and HFH in patients with an admission hs-cTnT value below the median (blue line) and admission hs-cTnT value above the median (green line) in the chronic HF subgroup (**C**). Survival analysis regarding the occurrence of worsening HF events and admission hs-cTnT values below the median (blue line) and above the median (green line) in the HFmrEF/HFpEF subgroup (**D**).

Kaplan–Meier survival analysis (Figure 3A,B) demonstrated that patients with admission hs-cTnT levels above the median value and patients with an increase in hs-cTnT during in-hospital stays experienced more commonly the composite outcome CV death and HFH (log-rank *p*-value = 0.02 and *p*-value = 0.03, respectively).

Cox regression analysis showed that an admission hs-cTnT above the median and an in-hospital increase in hs-cTnT represent an independent predictor of the composite of CV death and HFH at 6-month follow-up (Table 4 and Table 5).

The subgroup analysis, considering patients with chronic HF, demonstrated that the risk of the composite of CV death and HFH was significantly higher in patients with an admission hs-cTnT above the median value compared to patients with an admission hs-cTnT below the median value (7 vs. 2; 14.9% vs. 3.3%; *p* = 0.04) (Table 6).

Kaplan–Meier survival analysis evidenced that an admission hs-cTnT above the median value in the subgroup of patients with chronic HF is associated with an increased risk of CV death and HFH (log rank *p* = 0.03) at 6-month follow-up (Figure 3C).

The baseline features of patients according to LVEF values have been reported in Table 7.

Patients with HFmrEF/HFpEF and admission hs-cTnT above the median value had a significantly higher risk of outpatient WHF (i.e., urgent ambulatory visit/loop diuretic escalation) at 6-month follow-up compared to patients with HFmrEF/HFpEF and admission hs-cTnT below the median value (8 vs. 3; 33.3% vs 10%; *p* = 0.04) (Table 8).

Kaplan–Meier survival analysis demonstrated that patients with HFmrEF/HFpEF and an admission hs-cTnT above the median value have a significantly increased risk of experiencing an outpatient WHF event at 6-month follow-up (log rank *p* = 0.03) (Figure 3D).

## 4. Discussion

The identification of prognostic and predictive biomarkers is currently one of the biggest challenges for the improvement of HF management. The only validated biomarkers in HF are NPs. Beyond NPs, the most promising biomarkers are hs-cTnT and suppression of tumorigenesis-2 ligand (sST2L), and both have been shown to be independent predictors of mortality in HF [17]. However, data regarding hs-cTnT as prognostic tool in HF are discordant and often confusing, as well as scarce [18].

The results of our study highlighted the role of hs-cTnT as a valid prognostic biomarker in the total population of HF patients. More specifically, our results demonstrated that not only admission hs-cTnT values above the median, but also hs-cTnT increase during hospitalization are independent predictors of the composite of CV death and HFH at 6-month follow-up (OR: 2.06; 95% CI: 1.02–4.1; *p* = 0.04 and OR:1.95; 95%CI: 1.006–3.769; *p* = 0.04, respectively).

Previous studies revealed the possible role of troponins as predictive biomarkers of major CV events in HF patients [19,20,21,22,23,24,25,26,27,28,29]. Latini et al. [19] demonstrated that high levels of hs-cTnT were moderately associated with CV death in chronic HF patients, with a risk that was 5% higher when troponin levels above the median were detected. You et al. [20] identified that cardiac troponin I (cTnI) was an independent predictor of all-cause mortality in patients with acute decompensated HF. These results were confirmed in different studies including cTnI [21,22,23]. Del Carlo et al. [24] demonstrated a higher incidence of 1-year rehospitalization due to HF and mortality in patients with persistent troponin T levels higher than 0.02 ng/dl. Aimo et al. [25] conducted a meta-analysis analyzing a global population of 9289 in which it was confirmed that cTnT was an independent predictor of all-cause mortality and CV hospitalizations in patients with chronic HF. In a meta-analysis by Masson et al. [26] including 5284 patients, hs-cTnT levels were predictors of cardiovascular events in patients with chronic HF; however, it did not add significant prognostic discrimination. In acute decompensated HF patients, Peacock et al. [27] conducted a retrospective analysis on a population of 84872 patients hospitalized due to acute HF decompensation. A higher in-hospital mortality for patients with elevated hs-cTnT levels at admission has been observed [27]. Furthermore, Pandey et al. [28] reported that cardiac troponin elevation in patients with acute decompensated HFpEF was a predictor of adverse in-hospital and post-discharge events. In a recent meta-analysis by Evans et al. [29] including 67063 patients, hs-cTnT was associated with incident HF, improving also HF prediction.

These results, including the results of our study, are supported by a physiological explanation. It is known that the blood concentration of cardiac troponins is a consequence of myocardial cell necrosis and that every clinical condition that causes cardiomyocyte damage is also a cause of cardiac troponin blood level elevation [18,30]. In HF patients, cardiac troponin release may happen as a consequence of chronic ischemia, also in the absence of acute coronary stenosis [31]. This is due to HF-induced myocardial remodeling and subendocardial ischemia, determined by the excessive myocardial wall stress and cardiomyocyte damage [31]. Also, increased filling pressures, tachyarrhythmia or bradyarrhythmia, arterial hypotension, anemia, and endothelial dysfunction may be reasons for reduced oxygen supply to cardiomyocytes [32]. The consequence is the generation of myocardial injury, with an increase in cell permeability, allowing cytosol troponin to be released into the circulation [33,34].

Also, anemia and iron deficiency are known comorbidities associated with adverse events and worse life quality in patients with HF [1]. According to the Guidelines and the World Health Organization, anemia is defined by a hemoglobin level < 12 g/dL and <13 g/dL in females and males, respectively [1]. In our population, hemoglobin represented a predictor of CV death/HFH at univariate analysis for the total population, but it did not reach statistical significance at multivariate analysis.

Most of the mentioned studies are limited to describing an association between troponins and major CV events [35], without considering subclinical events in WHF and HF subgroups. Scenarios of WHF without hospitalization, such as escalation of diuretic therapy and/or need for urgent ambulatory visits, are also important concerns in the management of HF patients. This aspect has been highlighted by the consideration that hospitalization can be compared to the “tip of the iceberg” of a complex process of disease-worsening, which occurs outside of the hospital and is often subclinical [4,5]. It has been demonstrated that outpatient escalation of diuretics therapy increases the risk of 1-year mortality by 75% [36]. WHF is a transversal condition which involves HF patients regardless of LVEF.

HFrEF is widely studied in the scientific literature, while HFmrEF and HFpEF are entities less studied, but growing evidence demonstrates that their prognoses are similar to HFrEF [37]. In our study, we found that patients with HFmrEF/HFpEF and an admission hs-cTnT above the median value had a significant higher risk of urgent ambulatory visit/loop diuretic escalation at 6 months compared to HFmrEF/HFpEF patients with hs-cTnT below the median value (*p* = 0.03). It is known that HFpEF and HFmrEF populations have substantial differences compared to HFrEF patients. HFpEF patients are usually older and have multiple comorbidities with a less frequent history of ischemic heart disease than in HFrEF [38]. Furthermore, HFpEF has a greater association with extracardiac comorbidities, as well as with the female gender [39]. Although the mortality is similar in HFpEF and HFrEF, there has been shown to be a higher incidence of hospitalization in HFpEF, which is mainly related to worsening comorbidities [40]. It has been shown that HFmrEF has more similar outcomes to HFpEF than HFrEF [41]. The population of HFmrEF, similarly to HFpEF, is composed of older people and has a higher comorbidity burden than the HFrEF population. The prognosis of HFmrEF and HFpEF is mainly influenced by the adverse events related to comorbidities, and the role of hs-cTnT in this patient population may be explained by continuous ventricular pressure overload with consequently subendocardial ischemia [33,42].

Another important result of our study was that the risk of the composite of CV death and HFH was significantly higher in patients with chronic HF and an admission hs-cTnT above the median value compared to patients with an admission hs-cTnT below the median value (*p* = 0.03). This finding emphasizes the prognostic significance of hs-cTnT levels at hospital admission in patients with HF. Chronic HF patients with elevated hs-cTnT levels are likely to have underlying cardiac damage or stress, predisposing them to a higher risk of adverse cardiovascular outcomes. The elevated hs-cTnT levels on admission serve as a marker of continuous myocardial injury [43] in chronic HF patients. Our results seem to suggest that the long-standing steady myocardial damage in chronic HF may severely impact the prognosis.

The use of biomarkers such as NPs and cardiac troponins is suitable in most hospitals and outpatient services, and their use to manage patients is feasible and standardizable. On the contrary, other biomarkers, albeit interesting, are not always available everywhere. NPs and cardiac troponins reflect two different pathophysiological pathways in HF [44,45], whose involvement may vary according to the HF subgroup considered. Therefore, the integrated evaluation of the latter may bring relevant information which can be integrated into clinical evaluation in light of better patient management. Importantly, our results highlight the potential role of hs-cTnT quantification, in order to predict adverse events in peculiar HF subpopulations. An emerging and challenging aspect is the possibility of using these biomarkers not only to stratify patients’ prognoses, but also to guide therapy with HF disease-modifying drugs in order to identify patients at higher risk of adverse events and be more aggressive with the up-titration of therapy [6,17,46,47]. This aspect has been recently evaluated for NPs, with promising results.

Our study has several limitations. The results should be confirmed on a larger population and larger subgroups of HF patients. Due to the number of patients, multivariate analysis has not been used for subgroups. Other biomarkers have not been included in the study and compared to hs-cTnT in terms of prognostic predictivity. The trend of hs-cTnT has not been evaluated during the follow-up.

## 5. Conclusions

The assessment of biomarkers in HF represents a crucial aspect of patient management. Our results suggest that in-hospital hs-cTnT levels may predict the composite of CV death/HFH in patients with HF. In particular, in the subgroup of chronic HF patients, hs-cTnT may predict the composite of CV death/HFH, while in HFmrEF/HFpEF subgroup, hs-cTnT may predict out of hospital WHF events. Our results emphasize the importance of the serial assessment of hs-cTnT at admission and during hospitalization to assess the prognosis in HF patients.

## Figures and Tables

**Table 1:** Baseline features of the study population at hospital admission.

Variable	Total Population (N = 253)
**Age, years (IQR)**	73 (64.5–80)
**Male sex, n (%)**	177 (70)
**Arterial hypertension, n (%)**	195 (77.1)
**Diabetes mellitus, n (%)**	72 (28.5)
**Dyslipidemia, n (%)**	133 (52.6)
**Family history of CVD, n (%)**	66 (26.1)
**COPD, n (%)**	67 (26.5)
**Smoking habit, n (%)**	96 (37.9)
**Ischemic, n (%)**	138 (54.5)
**Hypertensive, n (%)**	35 (13.8)
**Idiopathic, n (%)**	29 (11.5)
**Valvular, n (%)**	29 (11.5)
**Inflammatory/drug induced, n (%)**	22 (8.7)
**Acute presentation, n (%)**	146 (57.7)
**Chronic presentation, n (%)**	107 (42.3)
**eGFR, mL/min/m^2^ (IQR)**	64 (46–81.7)
**Hemoglobin, g/dL (IQR)**	12.9 (10.9–14.3)
**K^+^, mmol/L (IQR)**	4 (3.68–4.33)
**Admission hs-cTnT, ng/mL (IQR)**	0.031 (0.02–0.078)
**Discharge hs-cTnT, ng/mL (IQR)**	0.031 (0.02–0.077)
**hs-cTnT peak, ng/mL (IQR)**	0.042 (0.023–0.121)
**hs-cTnT delta peak-admission, ng/mL (IQR)**	0.001 (0–0.026)
**HFrEF, n (%)**	199 (78.7)
**HFmrEF/HFpEF, n (%)**	54 (21.3)
**LVEF, % (IQR)**	32 (25–40)
**LVEDD, mm (IQR)**	58 (52–64)
**IVS, mm (IQR)**	11 (9–12)
**PW, mm (IQR)**	10 (9–10.5)
**Basal RVEDD, mm (IQR)**	36 (31–44)
**TAPSE, mm (IQR)**	18 (15–20)
**Median NYHA, class (IQR)**	3 (2–3)

IQR—interquartile range; CVD—cardiovascular disease; COPD—chronic obstructive pulmonary disease; eGFR—estimated glomerular filtration rate; K^+^—potassium; hs-cTnT—high-sensitivity T troponin; HFrEF—heart failure with reduced ejection fraction; HFmrEF—heart failure with mildly reduced ejection fraction; HFpEF—heart failure with preserved ejection fraction; LVEF—left ventricular ejection fraction; LVEDD—left ventricular end diastolic diameter; IVS—interventricular septum; PW—posterior wall; RVEDD—right ventricular end diastolic diameter; TAPSE—tricuspid annular plane systolic excursion; NYHA—New York Heart Association.

**Table 2:** Relationship between high-sensitivity T troponin at admission and the occurrence of each outcome in the total population.

Variable	hs-cTnT below Median Value	hs-cTnT above Median Value	*p* Value
**CV death/HFH, n (%)**	13 (9.5)	23 (19.8)	0.02
**CV death, n (%)**	8 (5.8)	15 (12.9)	0.05
**HFH, n (%)**	9 (6.6)	11 (9.5)	0.4
**Urgent visit/loop diuretic escalation, n (%)**	21 (15.3)	20 (17.2)	0.68

CV—cardiovascular; HFH—heart failure hospitalization; hs-cTnT—high-sensitivity T troponin.

**Table 3:** Relationship between high-sensitivity T troponin increase during hospitalization and the occurrence of each outcome in the total population.

Variable	No hs-cTnT Increase	hs-cTnT Increase	*p* Value
**CV death/HFH, n (%)**	16 (10.5)	20 (20)	0.03
**CV death, n (%)**	10 (6.5)	13 (13)	0.08
**HFH, n (%)**	11 (7.2)	9 (9)	0.6
**Urgent visit/loop diuretic escalation, n (%)**	28 (18.3)	13 (13)	0.26

CV—cardiovascular; HFH—heart failure hospitalization; hs-cTnT—high-sensitivity T troponin.

**Table 4:** Univariate and multivariate analysis regarding the variables considered as predictors of the composite event in the total population. High-sensitivity T troponin above the median at admission represents an independent predictor of cardiovascular death and heart failure hospitalization at 6-month follow-up in patients hospitalized with a diagnosis of heart failure.

Univariate			
Variable	OR	95% CI	*p* Value
**hs-cTnT above median**	2.2	1.117–4.353	0.02
**Age**	1.01	0.986–1.044	0.33
**Male sex**	0.75	0.380–1.479	0.40
**ACS**	1.12	0.489–2.550	0.79
**Arterial hypertension**	0.65	0.322–1.328	0.24
**Diabetes mellitus**	1.64	0.838–3.201	0.15
**eGFR**	0.99	0.994–1.004	0.78
**LVEF**	0.99	0.950–1.012	0.21
**Hemoglobin**	0.88	0.768–1.015	0.08
**Multivariate**			
**Variable**	**OR**	**95% CI**	***p* value**
**hs-cTnT above median**	2.06	1.025–4.128	0.04
**Hemoglobin**	0.94	0.815–1.090	0.42

hs-cTnT—high-sensitivity cardiac troponin T; OR—odds ratio; CI—confidence interval; ACS—acute coronary syndrome; eGFR—estimated glomerular filtration rate; LVEF—left ventricular ejection fraction.

**Table 5:** Univariate and multivariate analysis regarding the variables considered as predictors of the composite event in the total population. High-sensitivity T troponin increase during hospitalization represents an independent predictor of cardiovascular death and heart failure hospitalization at 6-month follow-up in patients hospitalized with a diagnosis of heart failure.

Univariate			
Variable	OR	95% CI	*p* Value
**hs-cTnT increase**	2.02	1.05–3.908	0.035
**Age**	1.01	0.968–10.44	0.33
**Male sex**	0.75	0.380–1.479	0.40
**ACS**	1.12	0.489–2.550	0.79
**Arterial hypertension**	0.65	0.322–1.328	0.24
**Diabetes mellitus**	1.64	0.838–3.201	0.15
**eGFR**	0.99	0.994–1.004	0.78
**LVEF**	0.98	0.950–1.012	0.21
**Hemoglobin**	0.88	0.768–1.015	0.08
**Multivariate**			
**Variable**	**OR**	**95% CI**	***p* value**
**hs-cTnT increase**	1.95	1.006–3.769	0.04
**Hemoglobin**	0.92	0.803–1.061	0.26

hs-cTnT—high-sensitivity cardiac troponin T; OR—odds ratio; CI—confidence interval; ACS—acute coronary syndrome; eGFR—estimated glomerular filtration rate; LVEF—left ventricular ejection fraction.

**Table 6:** Occurrence of each outcome according to high-sensitivity T troponin at hospital admission in chronic HF subgroup.

Variable	hs-cTnT below Median Value	hs-cTnT above Median Value	*p* Value
**CV death/HFH, n (%)**	2 (3.3)	7 (14.9)	0.04
**CV death, n (%)**	1 (1.7)	4 (8.5)	0.17
**HFH, n (%)**	1 (1.7)	5 (10.6)	0.08
**Urgent visit/loop diuretic escalation, n (%)**	8 (13.3)	12 (25.5)	0.1

CV—cardiovascular; HFH—heart failure hospitalization; hs-cTnT—high-sensitivity T troponin.

**Table 7:** Baseline features and discharge therapy of patients according to left ventricular ejection fraction.

Variable	HFrEF (N = 199)	HFmrEF/HFpEF (N = 54)	*p* Value
**Age, years (IQR)**	72 (64–80)	76 (68–81)	0.081
**Male sex, n (%)**	147 (73.9)	30 (55.6)	0.009
**Ischemic etiology, n (%)**	113 (56.8)	25 (46.3)	0.21
**Arterial hypertension, n (%)**	156 (78.4)	39 (72.2)	0.339
**Diabetes mellitus, n (%)**	59 (29.6)	13 (24.1)	0.421
**Dyslipidemia, n (%)**	104 (52.3)	29 (53.7)	0.851
**Family history of CVD, n (%)**	52 (26.1)	14 (25.9)	0.976
**COPD, n (%)**	51 (25.6)	16 (29.6)	0.554
**Smoking habit, n (%)**	75 (37.7)	21 (38.9)	0.872
**Acute presentation, n (%)**	120 (60.3)	26 (48.1)	0.024
**Chronic presentation, n (%)**	79 (39.7)	28 (51.9)	0.024
**eGFR, mL/min/m^2^ (IQR)**	63 (44–80)	66.3 (50–84.3)	0.62
**Hemoglobin, g/dL (IQR)**	13 (10.9–14.3)	12.5 (11.2–14.2)	0.46
**K^+^, mmol/L (IQR)**	4 (3.7–4.3)	4 (3.4–4.4)	0.55
**Admission hs-cTnT, ng/mL (IQR)**	0.031 (0.020–0.089)	0.031 (0.019–0.067)	0.817
**Discharge hs-cTnT, ng/mL (IQR)**	0.030 (0.020–0.074)	0.04 (0.02–0.079)	0.139
**hs-cTnT peak, ng/mL (IQR)**	0.04 (0.024–0.118)	0.044 (0.022–0.183)	0.852
**hs-cTnT delta peak-admission, ng/mL (IQR)**	0.001 (0–0.024)	0.003 (0–0.048)	0.375
**LVEF, % (IQR)**	30 (21–35)	45 (45–50)	<0.001
**LVEDD, mm (IQR)**	60 (54–65)	50.5 (45–56)	<0.001
**IVS, mm (IQR)**	11 (9–12)	11 (10–12.3)	0.077
**PW, mm (IQR)**	10 (9–11)	10 (9–10)	0.737
**Basal RVEDD, mm (IQR)**	34 (29–41)	38 (33–44)	0.1
**TAPSE, mm (IQR)**	18 (14–20)	19 (17–20)	0.029
**ACEi, n (%)**	17 (8.5)	17 (31.5)	<0.001
**ARBs, n (%)**	14 (7)	4 (7.4)	1
**ARNI, n (%)**	137 (68.8)	15 (27.8)	<0.001
**BB, n (%)**	189 (95)	53 (98.1)	0.466
**MRAs, n (%)**	165 (82.9)	29 (53.7)	<0.001
**SGLT2i, n (%)**	108 (54.3)	17 (31.5)	0.009
**Loop diuretics, n (%)**	153 (76.9)	31 (57.4)	0.004
**Median NYHA, class (IQR)**	3 (2–3)	3 (2–3)	1

IQR—interquartile range; CVD—cardiovascular disease; COPD—chronic obstructive pulmonary disease; eGFR—estimated glomerular filtration rate; K^+^—potassium; hs-cTnT—high-sensitivity T troponin; HFrEF—heart failure with reduced ejection fraction; HFmrEF—heart failure with mildly reduced ejection fraction; HFpEF—heart failure with preserved ejection fraction; LVEF—left ventricular ejection fraction; LVEDD—left ventricular end diastolic diameter; IVS—interventricular septum; PW—posterior wall; RVEDD—right ventricular end diastolic diameter; TAPSE—tricuspid annular plane systolic excursion; ACEi—angiotensin-converting enzyme inhibitor; ARBs—angiotensin receptor blockers; ARNI: angiotensin receptor/neprilysin inhibitor; BB: beta blocker; MRA—mineralocorticoid receptor antagonist; SGLT2i—sodium glucose cotransporter 2 inhibitor; NYHA—New York Heart Association.

**Table 8:** Occurrences of each outcome in the subgroup of patients with HFpEF/HFmrEF according to hs-cTnT values at hospital admission.

Variable	hs-cTnT below Median Value	hs-cTnT above Median Value	*p* Value
**CV death/HFH, n (%)**	2 (6.7)	6 (25)	0.12
**CV death, n (%)**	1 (3.3)	3 (12.5)	0.31
**HFH, n (%)**	1 (3.3)	4 (16.7)	0.16
**Urgent visit/loop diuretic escalation, n (%)**	3 (10)	8 (33.3)	0.04

CV—cardiovascular; HFH—heart failure hospitalization; hs-cTnT—high-sensitivity T troponin.

## Data Availability

Data are available upon reasonable request.
